# Promoting caring communities through collective action

**DOI:** 10.1002/ajcp.70030

**Published:** 2025-12-02

**Authors:** Yolanda Suarez‐Balcazar

**Affiliations:** ^1^ Department of Occupational Therapy University of Illinois Chicago Chicago Illinois USA

**Keywords:** caring communities, care justice, collective action

## Abstract

Current times call for the promotion of caring communities through collective action. In caring communities, we feel connected, experience a sense of belonging, and deeply care about each other's health and well‐being. Seymour Sarason's notion of social change as the creation of settings may have anticipated the art and practice of creating caring communities. Caring communities are thriving environments where members are there for one another, provide support when experiencing challenges, identify issues, take action on what matters to them, and celebrate achievements. In this paper, I call for creating caring communities through collective action. I discuss the concept of caring communities and propose a framework that introduces three key dimensions that characterize care‐inducing settings: mutual aid, building on community strengths and assets, and opportunities for engagement and shared power. I discuss how together, these dimensions foster a deep sense of community and belonging, the adoption of caring practices and advocacy, and promote voice, influence, and meaningful social participation. This framework invites a reimagining of community as a relational and justice‐oriented space where caring is not peripheral but central to how we organize, engage, and transform together. Examples of my research and action scholarly work illustrate the framework.

## Highlights

- Carrying communities promote sense of community and sense of belonging.
- Caring communities are thriving environments where members are there for one another, provide support when experiencing challenges, and together identify issues, and take action.
- Key dimensions that characterize care‐inducing settings include mutual aid, building on community strengths and assets, and opportunities for engagement and shared power.

*The greatness of a community is most accurately measured by the compassionate actions of its members.*

Coretta Scott King

The role of community psychology is more relevant today than ever, particularly considering the current political climate. As we celebrate the 20th SCRA Biennial and reflect on the 2025 conference theme, we aim to reach *“*new heights*”* in these challenging times. The challenges we face as a country deeply impact the issues we care about: sense of community and belonging, diversity, equity, and inclusion (DEI), disparities in health and well‐being, and social and economic inequalities. The depth and breadth of these inequities are becoming strikingly evident. They are further exacerbated by attacks on DEI, higher education, science, migrant communities, and many other groups that have experienced marginalization. Our core values and principles as community researchers and practitioners are under threat. The times call for the promotion of *caring communities through collective action*. In caring communities, we feel connected, experience a sense of belonging, and deeply care about each other's health and well‐being. While individual acts of caring—people looking out for one another—have always existed in our communities, today's sociopolitical climate makes such acts more difficult. Messages suggesting that “the other or they” are not worthy of compassion undermine this practice and threaten our shared humanity.

The title of the 20th Biennial Plenary at Michigan State University, “Meeting the Moment: Community Psychology in the Current Political and Economic Landscape,” reminds us that our values are increasingly caught in divisive political and populist rhetoric. Sadly, the growing discourse of “us” versus “them” or the “other” is becoming normalized, as reflected in recent national and global events. These trends are clear examples of exclusionary politics. This language of division promotes exclusion and further marginalizes groups that have historically faced oppression—including migrant communities, LGBTQ individuals, people of color, and people with disabilities, among others. Rather than fostering inclusive and caring communities, the current rhetoric reinforces separation and division, perpetuating racism, sexism, xenophobia, ableism, and broader patterns of hate and exclusion.

In 1974, Sarason wrote in *The Psychological Sense of Community*: “The thing we can be certain about is that in our society the absence or dilution of the psychological sense of community is the most destructive dynamic in the lives of people in our society” (Sarason, 1974, p. viii).

Today's rhetoric threatens to dilute and divide us—and in some cases, to dismantle our sense of community altogether. Dominant narratives often reinforce a narrow and exclusive definition of community, segmenting people by immigration status, gender identity, race, ethnicity, religion, ability, and other characteristics. These divisions are not only ideological; they reflect real social problems perpetuating exclusive forms of belonging. Given the current climate, now more than ever, we must commit to fostering caring communities and embracing the power of collective action. As community researchers and practitioners, we are uniquely positioned to counter these harmful narratives by engaging in research and action that builds inclusive, supportive communities. This paper calls for a renewed focus on creating caring communities. I begin with my personal background, followed by a conceptual framework for caring communities, illustrated through my community research and action scholarship. I conclude with recommendations for action.

## EARLY INTRODUCTION TO CARING COMMUNITIES AND THE POWER OF COLLECTIVE ACTION

### My personal background

My early exposure to caring communities began in childhood, growing up in Bogotá, Colombia, in a large, close‐knit family with 11 siblings. From a young age, we learned to care deeply for each other's health and well‐being and those around us—especially the most vulnerable, such as children living on the streets of Bogotá and low‐income families.

One formative example, out of several, of this value in action came when my parents learned about a small, rural village in the mountains on the outskirts of Bogotá. The village had recently constructed a school building, but months had passed without any teachers being hired, leaving children waiting eagerly for classes to begin. Motivated by the principles of Liberation Theology (Gutiérrez, 1988)—specifically the call for a *preferential option for the poor* and the message to act *here and now*—my parents decided to take action. Though neither were formally trained educators, they quickly organized their own “troop” of children (us) to step in as volunteer teachers. On weekends, we became instructors—my mother acted as the principal and lead teacher, while my father served as the administrator. All this was done voluntarily while we remained students during the week. Over time, local families began to rally around the school, forming a caring community united by a shared goal: ensuring their children could access education. This momentum led to a collective advocacy effort. Alongside a few families, we marched to City Hall, carrying signs and giving speeches to request hiring professional teachers. After several days of advocacy, the village responded—the school officially opened with qualified staff, and the need for 12–17‐year‐old “teachers,” organized by their parents, came to an end.

Years later, in the early 1980s, I joined Steve Fawcett's research team at the University of Kansas (KU) for graduate studies. There, I was introduced to several community research and action initiatives that embodied caring communities and the power of collective action. One such initiative involved a collaboration with the disability community. We used a participatory, action‐oriented, needs assessment approach—the *Concerns Report Method*—developed by the KU colleagues, to identify priority issues, develop action plans, and engage in advocacy around accessibility for people with disabilities and other issues that matter to them (Fawcett et al., 1988; Suarez‐Balcazar et al., 1988). Our work emphasized the impact of inclusive community action and the role of research in driving meaningful change.

## A FRAMEWORK FOR PROMOTING CARING COMMUNITIES

In the next section, I will address two critical areas, define and explain caring communities, and propose a framework for promoting care‐inducing settings. Furthermore, will illustrate the framework through my community research and action projects.

### What are caring communities?

The art and practice of creating caring communities may have been anticipated by Sarason's concept of social change as the creation of settings (Sarason, 1972). Caring communities are thriving environments where members support one another, feel included, and have a strong sense of belonging (Allen et al., 2021; Suarez‐Balcazar et al., 2023). People are welcomed and valued regardless of beliefs or identity (Hemminger et al., 2021; Kocaoğlu et al., 2022; Powell, 2012). In these spaces, individuals offer help during challenges, identify shared issues together, take collective action, and celebrate achievements (Powell & Chen, 2017).

Caring communities are rooted in shared values such as mutual support, celebrating similarities, and accepting and appreciating differences (Powell, 2012). They promote meaningful participation, uphold dignity and respect, encourage co‐creation, and advocate for Justice, Equity, Diversity, and Inclusion (JEDI) (Powell, 2012; Suarez‐Balcazar et al., 2023). These communities also foster liberation, co‐empowerment, occupational justice, and a deep sense of belonging and sense of community. As Sarason (1974) noted, a sense of belonging and a sense of community are fundamental to our identity and well‐being. Caring communities are social environments that cultivate engagement, connection, and security. Individuals prioritize each other's well‐being, nurturing a culture of compassion, support, and mutuality (McMillan & Chavis, 1986; O'Connor, 2013).

When harm arises, caring communities respond with collective protection and action. Caring communities are also central to community development efforts, where members work together to address shared challenges while maintaining care for one another (Kocaoğlu et al., 2022). Caring communities empower individuals through collective strength. These values are not new; they originate in Indigenous, Native values of caring for the land and one another and other non‐Western traditions worldwide (Menzies et al., 2024). In Latin America, for example, these values are passed down through generations—as my family has done. Furthermore, examples of caring communities might be abundant around us.

As Powell (2012) states, developing caring communities and a strong sense of belonging gives us a sense of shared identity, helps us feel connected and acknowledged as part of something bigger than ourselves, and builds on our shared values and diverse voices. Intentional creation of these communities demands a commitment to social justice. Caring communities—and the collective action they inspire—are powerful for dismantling systemic, community, or individual injustice (Powell, 2012; Suarez‐Balcazar et al., 2023).

### A framework for care‐inducing settings

Caring communities derive their strength from collective action. Yet, when power is exercised without care, it can give rise to domination, authoritarianism, and oppression—conditions that breed mistrust, fear, and resistance. The framework proposed here positions *caring* not as a byproduct of community life, but as its foundation and essence of their identity. In this model, caring is the core around which the community revolves, shaping its identity and guiding its actions.

Three key dimensions characterize care‐inducing settings:

- Mutual aid—reciprocal support that strengthens relationships and promotes interdependence.
- Build on community strength and assets—recognizing and building upon the community's existing capacities, talents, and resources.
- Opportunities for engagement and shared power—inclusive structures that enable active participation, co‐leadership, and decision‐making.

**Figure 1:**
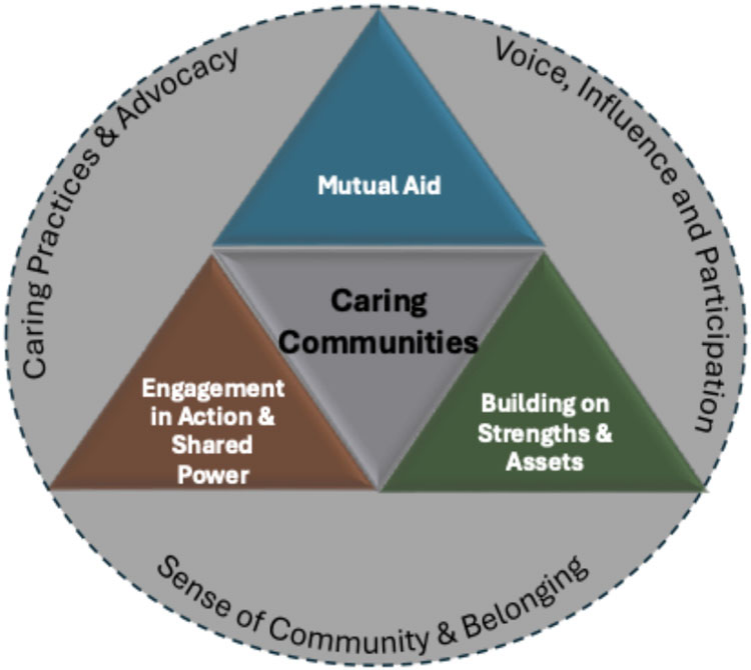
Care‐inducing settings framework.

These interconnected dimensions act as catalysts in cultivating caring communities. Together, they foster: a deep sense of community and belonging, the adoption of caring practices and advocacy, and voice, influence, and meaningful participation in society. This framework invites a reimagining of community as a relational and justice‐oriented space where care is not peripheral but central to how we organize, engage, and transform together. See Figure 1, A Framework for Care‐Inducing Settings.

The principles of caring communities are evident throughout the community psychology literature, though they are rarely labeled as such. For example, Latinx migrant families often build transnational networks where mutual care is the backbone of community life (Adames & Chavez‐Dueñas, 2017; Suarez‐Balcazar, 2020). Similarly, the Roma population—long marginalized across societies—has sustained strong family and community ties, drawing on their resilient cultural identity, they find civic purpose through everyday acts of caring and collective advocacy (Miranda et al., 2020). Native Indigenous communities have established forms of citizenship rooted in deep intergenerational ties to the environment, framing care for the land as an expression of community (see O'Keefe et al., 2025; Thompson‐Guerin & Mohatt, 2019). Additional examples include the work of Jason et al. (2016) with the Oxford House. The authors have demonstrated how communities of healing can engage in successful, community‐based interventions grounded in collective care. The CBPR work of Agner et al. (2020), in collaboration with the Clubhouse, is another example of caring communities. The Clubhouse promotes healing and a sense of community for individuals with mental illness by fostering caring communities, empowerment, and collective action. These examples, among others (e.g., O'Connor, 2013), underscore a central challenge: in an increasingly complex, divided and uncertain world, we must urgently reclaim and reimagine the meaning of community and belonging. We must recognize our interdependence and place care at the heart of organizing and sustaining community life.

While caring has traditionally been celebrated across cultures, it is often framed within gendered and maternal ideals—associated with kindness, love, and devotion. Yet, despite this cultural reverence, caregiving remains invisible mainly in political, economic, and social power structures. Suppose caring is to become a socially significant and transformative force. In that case, it must be reframed—not just as a personal virtue, but as a collective capacity for justice and systemic change (M. Garcia Ramirez, personal communication, June 12, 2025). As Dutt and Kohfeldt (2018) argue in *Towards a Liberatory Ethics of Care Framework for Organizing Social Change*, care must be viewed as a driver of social transformation. Their work helps us conceptualize *Care Justice*—whereby care is an active pursuit of equity and liberation, not simply a moral ideal. To this end, caring communities must become environments where the right to a dignified life is claimed, safeguarded, and championed. Public policy must reflect and uphold the value of care across all levels of society. In doing so, care becomes a form of civic participation—a vital force in collective action, social transformation, and the pursuit of justice.

## ADVANCING SOCIAL JUSTICE THROUGH CARING COMMUNITIES

### Exemplars from community research and action

*Alone we can do so little; together we can do so much*

Helen Keller

Advancing social justice demands more than preserving and building on past progress—it requires disrupting the status quo, engaging in advocacy, and amplifying the voices of historically marginalized communities. A renewed commitment to *social justice* and *care justice* calls for the intentional development of caring communities rooted in collective power, mutuality, and community‐led action. This transformative vision is best supported through community‐based participatory research (CBPR)—an approach grounded in equitable, mutually beneficial partnerships and shared purpose. CBPR is a strengths‐based and emancipatory process in which research and action are driven by community concerns and co‐developed by community members and researchers (Fawcett, 2021; Minkler, 2010; Suarez‐Balcazar et al., 2022). It emphasizes critical dialogue (Freire, 1973) and fosters meaningful engagement by co‐creating knowledge. (Suarez‐Balcazar, 2020). Since the first SCRA Biennial in 1987 at the University of South Carolina, community researchers and practitioners have contributed profoundly to advancing social justice, DEI, and sense of community and belonging. These contributions form the foundation of our collective progress. Yet today, that progress faces new threats. We cannot afford to retreat in a climate of rising polarization, backlash, and inequity. It is our responsibility to carry the momentum forward. We must protect and deepen our shared values—not through passive preservation but active transformation. Now, more than ever, we must champion caring communities as spaces of resistance, empowerment, and collective action. In doing so, we reimagine caring not only as a private act of kindness, but as a public force for justice—one that affirms dignity, advances equity, and strengthens our commitment to shared liberation and justice (M. Garcia Ramirez personal communication, June 12, 2025).

#### Case examples of caring communities and the power of collective action

Grounded in community psychology values and principles—and influenced by the training I received from Steve Fawcett at the University of Kansas and the innovations of the KU Workgroup (see the Community Toolbox: https://ctb.ku.edu)—my community research and action work has focused on understanding the social determinants of health impacting Latinx families raising a child with a disability. A central aim has been to design culturally tailored interventions that promote health, well‐being, and community belonging while addressing unmet needs.

### Background on latinx immigrant families of children with disabilities

Latinx immigrant families of children with disabilities face multiple, intersecting challenges that negatively impact their health, well‐being, and quality of life (Zeng et al., 2025). Evidence shows that these families experience poor health outcomes, high levels of mental stress, and social isolation, among other challenges. In one study involving 105 Latina caregivers of children with intellectual and developmental disabilities (IDD), the authors reported that 44% lacked health insurance. They also found that “family cohesion and social support were associated with greater maternal quality of life and family functioning” (Zeng et al., 2025, p. 8). In another study, focus groups conducted with Latinx youth with disabilities and their parents revealed a lack of access to culturally and linguistically relevant community‐based recreational and leisure programming for the entire family (Suarez‐Balcazar et al., 2018a).

#### Developing care‐inducing settings through community research and action

Over the past 20 years, my colleagues, students, and I have built a strong, reciprocal partnership with several community‐based organizations serving people with disabilities and their families in a predominantly Latinx neighborhood in Chicago. Following a series of successful, federally funded, community‐engaged research and action projects, we secured support from the Chicago Community Trust to codevelop a culturally and linguistically relevant community health promotion initiative. Community members and agency staff had identified this area of focus as a critical need.

Grounded in the Social Ecological Model (McLeroy et al., 1988) and guided by a Community‐Based Participatory Research (CBPR) approach, we co‐created a health initiative titled *Familias Saludables* (Healthy Families) (Suarez‐Balcazar et al., 2018b). The voices and leadership of the community—particularly a working group that included two Latina immigrant mothers, local caregivers, and agency staff were critical in developing the intervention. Together, we designed the program, which was piloted and sustained by the agency for several years before the COVID‐19 pandemic. *Familias Saludables* offered evidence‐based programming on health education, nutrition, physical activity, social learning, goal setting, and navigating the environment to promote health and well‐being. With additional support from the National Institute on Disability, Independent Living, and Rehabilitation Research (NIDILRR), we scaled up the initiative by integrating it with an existing intervention—*Caring for Myself* (Magaña et al., 2015)—and trained promotoras (community health workers) to deliver the new program called *PODER Familiar* (see Pei‐Lung et al., 2025; Suarez‐Balcazar et al., 2025).

#### Illustrating the framework components

*Mutual Aid*. Caring communities are rooted in mutual aid—where members offer one another consistent support, fostering trust, compassion, and resilience. In the Familias Saludables program, Latinx immigrant families often gathered in multigenerational groups. The in‐person weekly sessions quickly became a space for social learning, emotional connection, and community building. Participants created walking groups, initiated Zumba classes, exchanged resources, and offered mutual support to each other. In alignment with Sarason's concept of the creation of settings (Sarason, 1972), they co‐constructed a care‐inducing environment grounded in cultural values and collective well‐being.

*Building on Community Strengths and Assets*. Program implementation and conversations with families revealed a need to identify community resources and services for families raising children with disabilities. In response, we engaged in a community asset mapping process, co‐designed with two mother leaders from the group. Twenty‐one families participated in the mapping phase, followed by an open forum attended by 30 families to discuss findings. Later, a town hall meeting with a state representative drew 40 participants, who shared community assets and unmet needs (Suarez‐Balcazar et al., 2021b). Consistent with the literature (see Adames & Chavez‐ Duenas, 2017), key assets included a local faith‐based organization that provided culturally tailored programming to meet the needs of the Latinx community and bilingual healthcare providers knowledgeable about disability. Families also emphasized individual assets: their perseverance, resilience, and strong work ethic. As families identified resources, they organically expanded their networks—offering to introduce others to services, exchanging contact information, and attending events together, thus strengthening the fabric of the caring community (Suarez‐Balcazar et al., 2021b).

*Engagement in Action and Shared Power*. A defining feature of caring communities is the collective engagement in decision‐making and advocacy rooted in shared values. Families in the program began initiating new ideas and advocating for their needs. At their request, we co‐created a bilingual directory of culturally relevant community resources, which was widely distributed. When a local agency announced a meeting with a state representative, families requested time on the agenda to present their community concerns and assets. We supported their preparation by hosting practice sessions and encouraging their leadership. Community members took center stage at the public hearing—sharing their experiences, concerns, and visions for change. We remained in the background as they confidently led the meeting. Several parents emerged as powerful advocates, representing their community with pride and purpose. Later, they organized a public walk during a local health fair to raise awareness about pedestrian safety for youth with intellectual and developmental disabilities—responding to community concerns about unsafe traffic conditions. They created educational signs and distributed index cards, engaging the broader community in a conversation about accessibility and inclusion (Suarez‐Balcazar et al., 2021a). Through this study, families exercised shared power, strengthened their civic presence, and embodied the principles of a caring, justice‐oriented community.

### Sustaining caring communities

Planning for the sustainability of *Familias Saludables* included training local agency staff and mothers—many of whom emerged as leaders—to deliver the intervention with our ongoing support as needed. While the agency had intended to continue the program independently, its efforts were disrupted by the COVID‐19 pandemic. Fortunately, in collaboration with colleagues from the University of Texas at Austin, we secured funding from the National Institute on Disability, Independent Living, and Rehabilitation Research (NIDILRR) to scale up the initiative and continue addressing health disparities experienced by Latinx families raising children with disabilities. In this next phase, we co‐designed a new initiative titled *PODER Familiar*—a promotora‐delivered health and well‐being intervention that builds on *Familias Saludables* described above. The development of *PODER Familiar* was guided by ongoing input from two community advisory boards (Texas and Illinois) and regular feedback from our trained promotoras (community health workers). In Spanish, *PODER* means “power,” capturing the initiative's focus on empowering caregivers.

This study represents the culmination of several community‐engaged, action‐oriented projects focused on people living at the intersection of disability and ethnic minority status. As such, it offers a significant and distinctive contribution to the broader health equity research agenda. *PODER Familiar* specifically supports Latina immigrant caregivers of children with IDD (Pei‐Lung et al., 2025). Extensive research underscores the positive impact promotoras have on the health outcomes of Latina caregivers (Magaña et al., 2015). In our program, promotoras delivered the culturally tailored intervention through 10 one‐on‐one sessions held via Zoom. Participants were also invited to attend three group sessions hosted by the research team. The ten sessions focused on various aspects of health, including: caregivers' health and well‐being, healthy eating routines, physical activity, navigating the environment and decision‐making (Pei‐Lung et al., 2025). The promotoras, who were themselves Latina mothers of children with IDD, served as authentic change agents. Caregivers expressed that the promotora helped them create a community of support and mutual aid they didn't know existed—or that they had access to. To date, we have completed a pilot study (see Pei‐Lung et al., 2025) and are currently analyzing data from a randomized controlled trial. The trained promotoras supported families in navigating healthcare and wellness strategies and model community‐building, mutual support, and culturally grounded advocacy. Their efforts have helped caregivers strengthen social ties, acquire essential health knowledge, and build collective resilience. Looking ahead, sustainability efforts are being carried forward by our grassroots community partners, who have committed to adopting and offering the program to their participants. One such partner—an advocacy and support group founded and led by Latinx parents of children with disabilities—has grown into a vibrant, caring community. Their work reflects the key principles of mutual aid, community asset‐building, and engagement in collective action. Participation in these initiatives has fostered a deep sense of belonging, caring, and civic engagement. The ripple effects—stronger communities, empowered caregivers, and sustained advocacy—are a testament to the impact of cultivating caring communities.

## MOVING FORWARD DURING CHALLENGING TIMES

In today's climate—when so much is at stake—we need caring communities and the power of collective action. Below are some broader strategies we can use to foster widespread caring and create lasting change at the community level.

### Creating and sustaining care‐inducing settings

Widespread caring is urgently needed to support and protect communities that are being harmed. Since early in 2025, members of our migrant communities have experienced fear, anguish, and distress due to ongoing attacks, detentions, deportations, and threats of family separation. Our Latinx community—comprised of hardworking individuals—continues to be targeted and further marginalized. Many nationwide have joined Rapid Response Teams and sanctuary movements to support communities under attack. These teams are dedicated to building caring communities and offering support during moments of vulnerability. Training is provided to assist during community gatherings and to help safeguard spaces such as faith‐based settings and schools when a visible line of solidarity is necessary. One vital aspect of these efforts involves compiling a directory of agencies, community resources, and community assets that can be activated when a family faces trauma. These assets include individuals willing to offer emotional or spiritual support, legal assistance, temporary relief from food or housing insecurity, and more.

### Standing for JEDI: The power of storytelling

In a time when tactics are being used to silence diverse voices, some institutions have chosen to remove DEI content from websites and halt related initiatives, while others are strategically examining ways to continue to support DEI efforts and are steadfast in their commitment to equity and racial and social justice. Recently, I led a project titled *Advancing racial equity through elevating diverse voices: ARE Voices*. Alongside faculty, graduate students, and community partners, we launched an action‐oriented initiative to center strength‐based narratives of people from historically marginalized communities—those living with chronic conditions, disabilities, or caregiving experiences. In the face of harmful rhetoric that dehumanizes marginalized groups, we sought to amplify stories of resilience, spirituality, family and kinship, and advocacy through storytelling. Our storytellers often expressed how empowering it has been to have their voices heard—not in terms of deficits, but as a reflection of their strengths. Listening to their stories has been transformative to us as well. Some of our storytellers have emerged as advocates for disability rights and leaders in building caring communities. Our digital library of stories will be used to educate students, foster conversations, and guide research efforts from a strength‐based approach.

### Building on shared values

In times of deep polarization, help others articulate a vision of the world they would like to envision by facilitating a discussion on core values. This interactive reflection can be conducted with community residents or students. Have individuals share their core values, especially with those they don't usually interact with or view as different from themselves, yet share work, community, or classroom space. Remarkably, despite differences in ethnicity, religion, political beliefs, backgrounds, and other dimensions, we consistently find shared values such as kindness and compassion, voluntarism and service, family and friendships, faith, and respect and dignity, among others (Shetty, 2020). Once individuals share their values, they create action statements on how they want to enact them in their daily lives and the space they share. Follow‐up reflection sessions should focus on how values are being enacted. Reflecting on shared values and progress towards enacting values can foster a sense of community and belonging. Yet, this exercise is not enough to create lasting change; ongoing conversations and efforts to create caring communities are much needed.

### Engaging in collective action and advocacy

A substantial body of work in community psychology has documented the power of collective advocacy. We must continue mobilizing at the community, local, state, and national levels. Being there for one another at the community and local levels fosters caring communities. Yet, collective action may also involve contacting legislators and state representatives, writing letters and op‐eds, becoming an ally, signing and circulating petitions, and supporting or joining community mobilizing efforts, among other actions. For tools and strategies, see the Community Tool Box on advocating for change, and SCRA policy resources https://scra27.org/resources/policy-resources/.

## CONCLUSION

The framework and examples shared here illustrate the transformative power of caring communities. These communities have a liberating force—they help us organize, reframe dominant narratives, elevate cultural traditions, and affirm our values, enhancing our sense of belonging. The psychology of liberation teaches us that dominant institutions and systems of oppression can be countered by the collective strength of people who care, act, and share power. As illustrated in these examples—and likely in the work many of you do—this strength lies in our ability to reimagine community, commit to justice, and to one another. In the spirit of Seymour Sarason and the principles upheld by the Society for Community Research and Action since the first Biennial in South Carolina in 1987, these turbulent times demand that we walk the talk. We must live the values of advocacy, activism, empowerment, and community care; we can all make a difference. This journey starts with us—we are all in this together.

## References

[ajcp70030-bib-0001] Adames, H. Y. , & Chavez‐Dueñas, N. Y. (2017). *Cultural foundations and interventions in Latino/a mental health*: History, theory and within group differences. Routledge/Taylor & Francis Group. 10.4324/9781315724058

[ajcp70030-bib-0002] Agner, J. , Barile, J. P. , Botero, A. , Cha, T. , Herrera, N. , Kaukau, T. M. , Nakamura, L. , & Inada, M. , Hawai'i Clubhouse Coalition . (2020). Understanding the role of mental health clubhouses in promoting wellness and health equity using Pilinahā—An indigenous framework for health. American Journal of Community Psychology, 66(3–4), 290–301. 32955119 10.1002/ajcp.12457

[ajcp70030-bib-0003] Allen, K. A. , Gray, D. L. , Baumeister, R. F. , & Leary, M. R. (2021). The need to belong: A deep dive into the origins, implications, and future of a foundational construct. Educational Psychology Review, 34(2), 1133–1156. 10.1007/s10648-021-09633-6 34483627 PMC8405711

[ajcp70030-bib-0004] Dutt, A. , & Kohfeldt, D. (2018). Towards a liberatory ethics of care framework for organizing social change. Journal of Social and Political Psychology, 6(2), 575–590. 10.5964/jspp.v6i2.909

[ajcp70030-bib-0005] Fawcett, S. B. (2021). A reflection on community research and action as an evolving practice. Behavior and Social Issues, 30(1), 535–544. 38624970 10.1007/s42822-021-00083-x PMC8589455

[ajcp70030-bib-0006] Fawcett, S. B. , Suarez de Balcazar, Y. , Whang‐Ramos, P. L. , Seekins, T. , Bradford, B. , & Mathews, R. M. (1988). The concerns report: Involving consumers in planning for rehabilitation and independent living services, American Rehabilitation (Vol. 14, pp. 17–19).

[ajcp70030-bib-0007] Freire, P. (1973). Education for critical consciousness. Seabury Press.

[ajcp70030-bib-0008] Gutiérrez, G. (1988). In C. Inda , & J. Eagleson Trans.; Rev. ed., A Theology of liberation: History, politics, and salvation. Orbis Books.

[ajcp70030-bib-0009] Hemminger, W. , Elliott, J. A. , Dewig, S. , Kramer, K. , Bredhold, W. , Hochwender, C. G. , Stratman, A. J. , Pope, J. , Rich, A. , & Kimbrough, R. C. (2021). Growing good: A beginners guide to cultivating caring communities. Indiana University Press.

[ajcp70030-bib-0010] Jason, L. A. , Stevens, E. , & Light, J. M. (2016). The relationship of sense of community and trust to hope. Journal of Community Psychology, 44(3), 334–341. 10.1002/jcop.21771 27087710 PMC4828033

[ajcp70030-bib-0011] Kocaoğlu, B. U. , Kocaoğlu, M. , & Phillips, R. (2022). Beyond recovery and resilience: Achieving caring communities. In M. A. Brennan , R. Phillips , N. Walzer , & B. D. Hales (Eds.), Community development for times of crisis: Creating caring communities (pp. 255–270). Routledge.

[ajcp70030-bib-0012] Magaña, S. , Li, H. , Miranda, E. , & Paradiso de Sayu, R. (2015). Improving health behaviours of Latina mothers of youths and adults with intellectual and developmental disabilities. Journal of Intellectual Disability Research, 59(5), 397–410. 24761812 10.1111/jir.12139

[ajcp70030-bib-0013] McLeroy, K. R. , Bibeau, D. , Steckler, A. , & Glanz, K. (1988). An ecological perspective on health promotion programs. Health Education Quarterly, 15(4), 351–377. 3068205 10.1177/109019818801500401

[ajcp70030-bib-0014] McMillan, D. W. , & Chavis, D. M. (1986). Sense of community: A definition and theory. Journal of Community Psychology, 14(1), 6–23.

[ajcp70030-bib-0015] Menzies, A. K. , Bowles, E. , McGregor, D. , Ford, A. T. , & Popp, J. N. (2024). Sharing indigenous values, practices and priorities as guidance for transforming human–environment relationships. People and Nature, 6(Issue 5), 2109–2125. 10.1002/pan3.10707.

[ajcp70030-bib-0016] Minkler, M. (2010). Linking science and policy through community‐based participatory research to study and address health disparities. American Journal of Public Health, 100(Suppl. 1), S81–S87. 10.2105/AJPH.2009.165720 20147694 PMC2837446

[ajcp70030-bib-0017] Miranda, D. E. , García‐Ramírez, M. , & Albar‐Marín, M. J. (2020). Building meaningful community advocacy for ethnic‐based health equity: The RoAd4HealthExperience. American Journal of Community Psychology, 66(3–4), 347–357. 10.1002/ajcp.12443 32696550

[ajcp70030-bib-0018] O'Connor, B. (2013). From isolation to community: Exploratory study of a sense‐of‐community. Journal of Community Psychology, 41(8), 973–991. 10.1002/jcop.21587 31007302 PMC6474243

[ajcp70030-bib-0019] O'Keefe, V. M. , Maudrie, T. L. , Grubin, F. , Gonzalez, M. B. , Saniguq Ullrich, J. , Crouch, M. , White, E. , Desjardins, M. M. , Martin, L. , Lewis, M. , HorseChief, M. , Fernandez, M. , Phoebe Keryte, A. , & Walls, M. L. (2025). Someday, I'll be an ancestor. Understanding indigenous intergenerational connectedness through qualitative research to inform measure development American Journal of Community Psychology, early viewing, 76, 110–120. https://onlinelibrary.wiley.com/authored-by/White/Evan 10.1002/ajcp.12803 PMC12404140 40079177

[ajcp70030-bib-0020] Pei‐Lung, Y. , Suarez‐Balcazar, A. , Errisuriz, Y. , Parra‐Medina, V. L. , Mirza, D. , Zhang, M. , Pei‐Chiang Lee., M. , Zeng, W. , Brown‐Hollie, J. P. , Yespica Mendoza, E. , Brown, S. V. , Heydarian, S. B. , & Magana, S. (2025). PODER Familiar: A culturally tailored health intervention for Latino families of children with intellectual and developmental disabilities. Journal of Applied Research in Developmental Disabilities, 38(2), e70048. 10.1111/jar.70048 40186534

[ajcp70030-bib-0021] Powell, J. A. (2012). Racing to justice: Transforming our conceptions of self and others to build an inclusive society. Indiana University Press.

[ajcp70030-bib-0022] Powell, J. L. , & Chen, S. (2017). Care and trust: A new understanding. Health Education & Care, 2(4), 1–5. 10.15761/HEC.1000130

[ajcp70030-bib-0023] Sarason, S. B. (1972). The creation of settings and the future societies. Jossey‐Bass.

[ajcp70030-bib-0024] Sarason, S. B. (1974). The psychological sense of community: Prospects for a community psychology. Jossey‐Bass.

[ajcp70030-bib-0025] Shetty, J. (2020). Think like a Monk: Train your Mind for Peace and Purpose Every Day. Simon & Schuster.

[ajcp70030-bib-0026] Suarez‐Balcazar, Y. , Agudelo, A. , Arte, M. , & Garcia, C. (2018a). Unpacking barriers to healthy lifestyles from the perspective of youth with disabilities and their parents. Journal of Prevention and Intervention in the Community, 46(1), 61–72. 29281599 10.1080/10852352.2018.1386270

[ajcp70030-bib-0027] Suarez‐Balcazar, Y. , Arias, D. , & Muñoz, J. P. (2023). Promoting justice, diversity, equity, and inclusion through caring communities: Why it matters to occupational therapy. The American Journal of Occupational Therapy, 77(6), 7706347020. 10.5014/ajot.2023.050416 38015491

[ajcp70030-bib-0028] Suarez‐Balcazar, Y. , Bradford, B. , & Fawcett, S. B. (1988). Common concerns of disabled Americans: Issues and options. Social Policy, 19(2), 29–35. 10318229

[ajcp70030-bib-0029] Suarez‐Balcazar, Y. , Early, A. , Maldonado, A. , Garcia, C. , Arias, D. , Zeidman, A. , & Agudelo‐Orozco, A. (2018b). Community‐based participatory research to promote healthy lifestyles among Latino immigrant families with youth with disabilities. Scandinavian Journal of Occupational Therapy, 25(5), 396–406. 10.1080/11038128.2018.1502348 30280951

[ajcp70030-bib-0030] Suarez‐Balcazar, Y. , Early, A. , Miranda, D. , Arias, D. , Garcia, C. , Lopez, G. , & Gonzalez, J. (2021a). Latinx immigrant families of youth with disabilities participating in civic engagement to promote social justice. Social Justice Research Collaborative, I,1–29. 10.5210/sjrc.v0i0.11751

[ajcp70030-bib-0031] Suarez‐Balcazar, Y. , Early, A. , Miranda, E. D. , Marquez, H. , Maldonado, A. , & Garcia‐Ramirez, M. (2021b). Community‐engaged asset mapping with Latinx immigrant families of youth with disabilities. American Journal of Community Psychology, 65, 261–271. 10.1002/ajcp.12578 34963017

[ajcp70030-bib-0032] Suarez‐Balcazar, Y. , Pei‐Lung Yu, A. , Brown, S. , Brown, J. , Crostley, A. , Parra‐Medina, D. , Saenz, M. , Mirza, M. , Vanegas, S. , & Magana, S. (2025). Culturally tailored interventions: A conceptual model and case study to promote health and wellbeing of Latinx immigrant caregivers of children with disabilities. American Journal of Community Psychology, 75, 383–397. 10.1002/ajcp.12789 PMC12174727 39924993

[ajcp70030-bib-0033] Suarez‐Balcazar, Y. (2020). Meaningful engagement in research: community residents as co‐creators of knowledge. American Journal of Community Psychology, 65(3–4), 261–271. 10.1002/ajcp.12414 31907950

[ajcp70030-bib-0034] Suarez‐Balcazar, Y. , Balcazar, F. , Miranda, D. E. , Velazquez, T. C. , Garcia‐Ramirez, M. , & Arcidiacono (2022). Promoting justice through community‐based research: International case studies. American Journal of Community Psychology, 69(3–4), 318–330. 10.1002/ajcp.12584 35285953

[ajcp70030-bib-0035] The Community Tool Box . Also the CTB Chapter 28, section 2 on Being Compassionate, The content on building compassionate communities might be inspiring, and relevant to your piece. https://ctb.ku.edu/en/table-of-contents/spirituality-and-community-building/being-compassionate/main

[ajcp70030-bib-0036] Thompson‐Guerin, P. , & Mohatt, N. V. (2019). Community psychology and indigenous peoples. American Journal of Community Psychology, 64(1–2), 3–8. 10.1002/ajcp.12383 31489636

[ajcp70030-bib-0037] Zeng, W. , Suarez‐Balcazar, Y. , Errisuriz, V. L. , Mirza, M. , Vanegas, S. V. , Parra‐Medina, D. , & Magaña, S. (2025). Social determinants of health, maternal quality of life, and family functioning among Latinx families of children with intellectual and developmental disabilities. Journal of Autism and Developmental Disabilities, 1–13. 10.1007/s10803-025-06919-4. 40498256

